# The effects of Twinlight laser treatment on the titanium surface proliferation and osteogenic differentiation of mesenchymal stem cells

**DOI:** 10.1186/s12903-022-02448-z

**Published:** 2022-09-19

**Authors:** Mengzhen Zhao, Feng Qiu, Jianing Song, Congcong Zhang, Taohong Liu, Mingxuan Wu

**Affiliations:** 1 Hebei Key Laboratory of Stomatology, Department of Periodontology (II), Hebei Clinical Research Center for Oral Diseases, School and Hospital of Stomatology, Hebei Medical University, Zhongshan East Road 383, Shijiazhuang, 050017 Hebei People’s Republic of China; 2 Hebei Key Laboratory of Stomatology, Department of Laser Medicine, Hebei Clinical Research Center for Oral Diseases, School and Hospital of Stomatology, Hebei Medical University, Shijiazhuang, 050017 Hebei People’s Republic of China; 3 Hebei Medical University, Zhongshan East Road 361, Shijiazhuang, 050017 Hebei People’s Republic of China

**Keywords:** Twinlight laser, Mesenchymal stem cells (MSCs), Peri-implantitis, Osteogenesis, Lipopolysaccharide (LPS)

## Abstract

**Background:**

The study aimed to observe the effects of a Twinlight laser on the titanium surface proliferation of inflammatory Mesenchymal stem cells (MSCs), inflammatory cytokine expression, and osteogenic differentiation.

**Methods:**

The MSCs were collected from bone tissue of healthy individuals.The cellular inflammatory model was established with 1 μg/mL lipopolysaccharide (LPS).Under the cellular inflammatory model,divided into five groups: the normal control group (C); the inflammatory control group (L); Er:YAG laser group (L + E); Nd:YAG laser group (L + N); Er:YAG laser and Nd:YAG laser group (L + E + N). The treated cells were inoculated onto titanium disks.The normal and inflammatory MSCs on the surface of titanium surface were examined by CCK-8, scanning election microscopy (SEM), quantitative real-time polymerase chain reaction (qRT‑PCR) and other methods for their proliferation, growth pattern, expression of inflammatory factors Interleukin-6 (IL-6), Interleukin-8 (IL-8) and osteogenic genes Runx2 (Runt-related transcription factor 2) and alkaline phosphatase (ALP), providing the theoretical basis and experimental data for the Twinlight laser-assisted treatment of peri-implantitis. Statistical analyses were performed using a Student's t test with SPSS 17.0 software.

**Results:**

Through observation using SEM, the cell densities of the L + E + N, L + E, and L + N groups were similar, but cell bodies in the L + E + N group were fuller and each had more than two pseudopodia. The expression level of IL-6 mRNA in the L, L + N, L + E, and L + E + N groups was higher than in group C (P < 0.05), and the expression level of IL-8 mRNA in the L + E + N group was significantly lower than in group L (P < 0.0001). On day 7, the expression level of ALP mRNA in the L, L + N, L + E, and L + E + N groups was lower than in group C (P < 0.05). On day 14, there was no significant difference in the expression level of ALP mRNA among the L + N, L + E + N, and C groups (P > 0.05). On day 7, the expression level of RUNX2 mRNA in the L + E + N group was higher than in group L (P < 0.001). On day 14, the expression level of RUNX2 mRNA in the L + E + N group was higher than in group L (P < 0.01).

**Conclusion:**

Twinlight laser treatment promoted cell proliferation, inhibited the expression of inflammatory cytokines, and effectively enhanced the osteogenic differentiation of cells on a titanium surface.

## Introduction

Peri-implantitis refers to peri-implant lesions caused by dental plaque microorganisms, jaw wounds, and bone burns [1].The key to the treating is controlling dental plaque and promoting osseointegration and tissue regeneration by removing the implant, the surrounding microorganisms, and infected tissue [2].Traditional mechanical debridement and drug treatment can have some treatment effects; however, limitations remain in this regard, such as incomplete debridement, low work efficacy and implant surface damage during debridement [3]. Accordingly, how to effectively remove stimulants, eliminate inflammation, and promote osseointegration has developed as research topics.

Laser treatment can deliver ablation, vaporization, microbial inhibition, and biological effects, and can effectively improve peri-implant gum redness, swelling, and bleeding, and probe the signs of peri-implantitis without additional damage to surrounding soft, hard, and deep tissue types [4].

Erbium-doped-yttrium aluminum garnet (Er:YAG) and neodymium-doped (Nd:YAG) lasers present different advantages in terms of sterilization, anti-inflammation, removing infected soft and hard tissue, and promoting tissue healing [5].These lasers are considered to have the best potential in terms of laser therapy for peri-implantitis [6]. However, the combined application effect of lasers operating at different wavelengths requires additional study.

Mesenchymal stem cells (MSCs) are adult stem cells derived from oral alveolar bone and exhibit multiple differentiation potentials. Additionally, these cells can be separated into osteoblasts, adipocytes, and endothelial cells to contribute to the repair of hard and soft tissue around dental implants.

The present study aimed to investigate the roles of Twinlight laser in control inflammationand adipogenic differentiation of MSCs under inflammatory conditions.Additionally, the study aimed to establish a theoretical basis and experimental data for the clinical treatment of peri-implantitis.

## Methods

### Cell culture and identification

Fragments of peripheral bone tissue and buccolingual bony plates were collected in healthy individuals aged 18–35 years after the third molars were removed in the Department of Oral and Maxillofacial Surgery in the Hospital of Stomatology, Hebei Medical University, China. The patients were informed of the sample material collection and signed an informed consent form permitting the researchers to do so. The study was approved by the Institutional Review Board of the Hospital of Stomatology, Hebei Medical University (approval no. [2021]003). Upon acquisition, the bone tissue was immediately soaked in an alpha minimum essential medium (α-MEM) containing a 2% penicillin–streptomycin (PS) solution. The bone tissue was rinsed 10 times with a phosphate-buffered saline (PBS) solution containing 2% PS. Next, the bone tissue was trimmed with tissue scissors until the volume was less than 1 mm^3^, and the obtained fine bone tissue was transferred to a centrifuge tube. To each tube, 3 ml of 0.1% collagenase was added, and the tube was shaken and digested at 37˚C for 50 min. Subsequently, 0.25% trypsin was added for further digestion for 25 min. Thereafter, a small volume of 10% FBS was added to terminate the digestion. Following centrifugation at 1000 × g for 10 min at room temperature, the supernatant was discarded and then inoculated into a culture flask [7]. Cell adherence and growth were observed 24 h later. MSCs were characterized by the detection of cell surface markers (CD29, CD44, and CD45)through flow cytometric analysis.

### Osteogenic induction, ALP and alizarin red staining

For osteogenic differentiation, MSCs were cultured in normal mediun with 1 μM dexamethasone, 100 μg/L ascorbic acid and 2 mmol/L sodium beta-glycerophosphate.After 7 days, MSCs were fixed with 70% alcohol, and add the ALP incubation media B1 and B2 (mixed in a 1:1 proportion) toeach well and incubate MSCs for 20 min at 37 ℃.After14 days,MSCs were fixed with 4% paraformaldehyde and stained with 2% Alizarin Red (pH = 4.2)for 10 min at 37 ℃.

### Cell proliferation

On days 1, 3, 5, and 7, the medium was removed, and the cells were rinsed 3 times. Then, 10 μl of the CCK-8 kit (庄盟, 中国) was added to each well,and cells were incubated at 37 °C for 2 h. The optical density at 450 nm was measured using aspectrophotometer, and the cell proliferation curve was plotted.

### Preparation of the laboratory reagents

The lipopolysaccharide (LPS) induction solution was created as follows: 10 mg LPS powder was dissolved in 10 ml PBS to create a 1 mg/ml stock solution, which was diluted to 1 μg/ml by the Complete medium for use in experiments and kept at –20 °C.

### Experimental design

For this investigation, 15-mm-diameter machined titanium were polished in -400, -600 and -1200 granulation bands and then cleaned with acetone,ethanol, and deionized water. Prior to cell seeding,disks were individually placed in wells of sterilized 24-well plates. The cells in the C group were cultured using a conventional medium; for the remaining four groups, cells were cultured in a 1-μg/ml LPS medium for 24 h; thereafter, conventional culture and medium change continued for the LPS group. In the LPS + Er:YAG laser group, the digested and centrifuged cells were placed in a centrifuge tube for Er:YAG laser irradiation (MSP, 60 mJ, 20 Hz, 1.2 W; water, 0; gas, 0), and the laser emission source was placed 10 cm above the center of the centrifuge tube for 60 s irradiation. For the LPS + Nd:YAG laser group, the digested and centrifuged cells were placed in a centrifuge tube for Nd:YAG laser irradiation (MSP, 15 Hz, 1.50 W) with a 300-μm optical fiber head, and the laser emission source was placed 10 cm above the center of the centrifuge tube for 60 s irradiation. For the LPS + Twinlight laser group, the digested and centrifuged cells were placed in a centrifuge tube for 120 s Twinlight laser irradiation (Er:YAG laser + Nd:YAG laser) using the same method. Following treatment, the cells were counted and inoculated onto the titanium disks [8, 9].

### Cell proliferation assay

On days 1, 3, 5, and 7, six samples were collected from each group, rinsed with a PBS solution 2–3 times, and transferred to a new 24-well plate. To each well, a 450-μl serum-free medium and a 50-μl CCK-8 solution were added, and cells were incubated at 37 °C for 2 h. The optical density at 450 nm was measured using aspectrophotometer, and the cell proliferation curve was plotted.

### Scanning electron microscope

Following irradiation, the cells in the five groups were counted, respectively, and inoculated onto a 24-well titanium disk with 5 × 10^3^ cells. 24 h later, the samples were rinsed with a PBS solution three times and then soaked in 0.1 mol/L PBS containing 2.5% glutaraldehyde for 1 h. Following fixation, the samples were rinsed with PBS three times (10 min each time). Then, the samples were separately dehydrated in 35%, 50%, 75%, 90%, and 100% ethanol for 15 min, and placed in an HCP-2 critical point dryer for vacuum drying. Following gold plating of the surface, the samples were placed under a scanning electron microscope (SEM) to observe the root surface morphology and the attachment of MSCs, and photos were taken.

### Quantitative real-time PCR (qRT-PCR)

MSCs were seeded onto titanium sheets in 24-well plates at 1 × 10^4^ cells after irradiation, and duplicate wells were set up for each group. 24 h later, osteogenic cultures were induced and the medium was changed every 3 days. The culture medium was discarded after 12 h and 24 h of culture and 7.14 d of osteogenic induction culture, and total RNA was extracted and reverse transcribed into cDNA according to the instructions of the Trizol kit. Associated gene primers were designed based on qRT-PCR. Synthesized primer sequences are shown in Table 1. Primers were synthesized by Sangon Biotech Co. Ltd. Expression of IL-6, IL-8, ALP, and RUNX2 was measured by qRT-PCR, with GAPDH as internal standards.

**Table 1:** Gene primer sequences used for qRT-PCR

Primer	Forward	Reverse
GAPDH	GGAGCGAGATCCCTCCAAAAT	GGCTGTTGTCATACTTCTCATGG
IL-6	AGACAGCCACTCACCTCTTCAG	TTCTGCCAGTGCCTCTTTGCTG
IL-8	GAGAGTGATTGAGAGTGGACCA	CACAACCCTCTGCACCCAGTTT
ALP	AACATCAGGGACATTGACGTG	GTATCTCGGTTTGAAGCTCTTCC
Runx2	CCCAGTATGAGAGTAGGTGTCC	GGGTAAGACTGGTCATAGGACC

*IL-6* Interleukin-6; *IL-8* Interleukin-8; *ALP* alkaline phosphatase; *RUNX2* Runt-related transcription factor-2

### Statistical analysis

Quantitative data are expressed as mean + standard deviation (SD). Statistical analyses were performed using a Student's t test with SPSS 17.0 software. P < 0.05 was considered statistically significant and shown with an asterisk in the bar diagrams.

## Results

### Isolation of MSCs

**Fig. 1:**
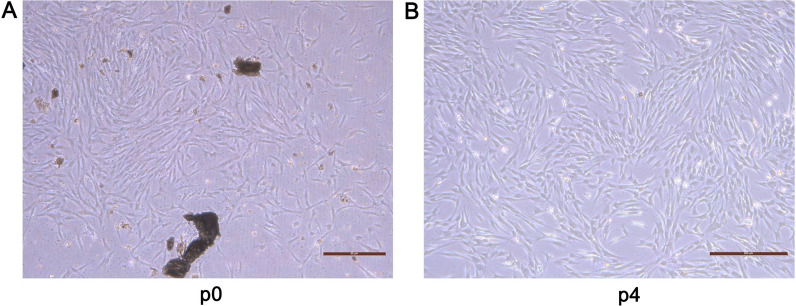
Isolation and culture of MSCs from tissue blocks. Primary MSCs sub‑cultured for **A** P0 and **B** P4. MSCs, MSCs; P, passage. (Inverted Microscope, × 100)

**Fig. 2:**
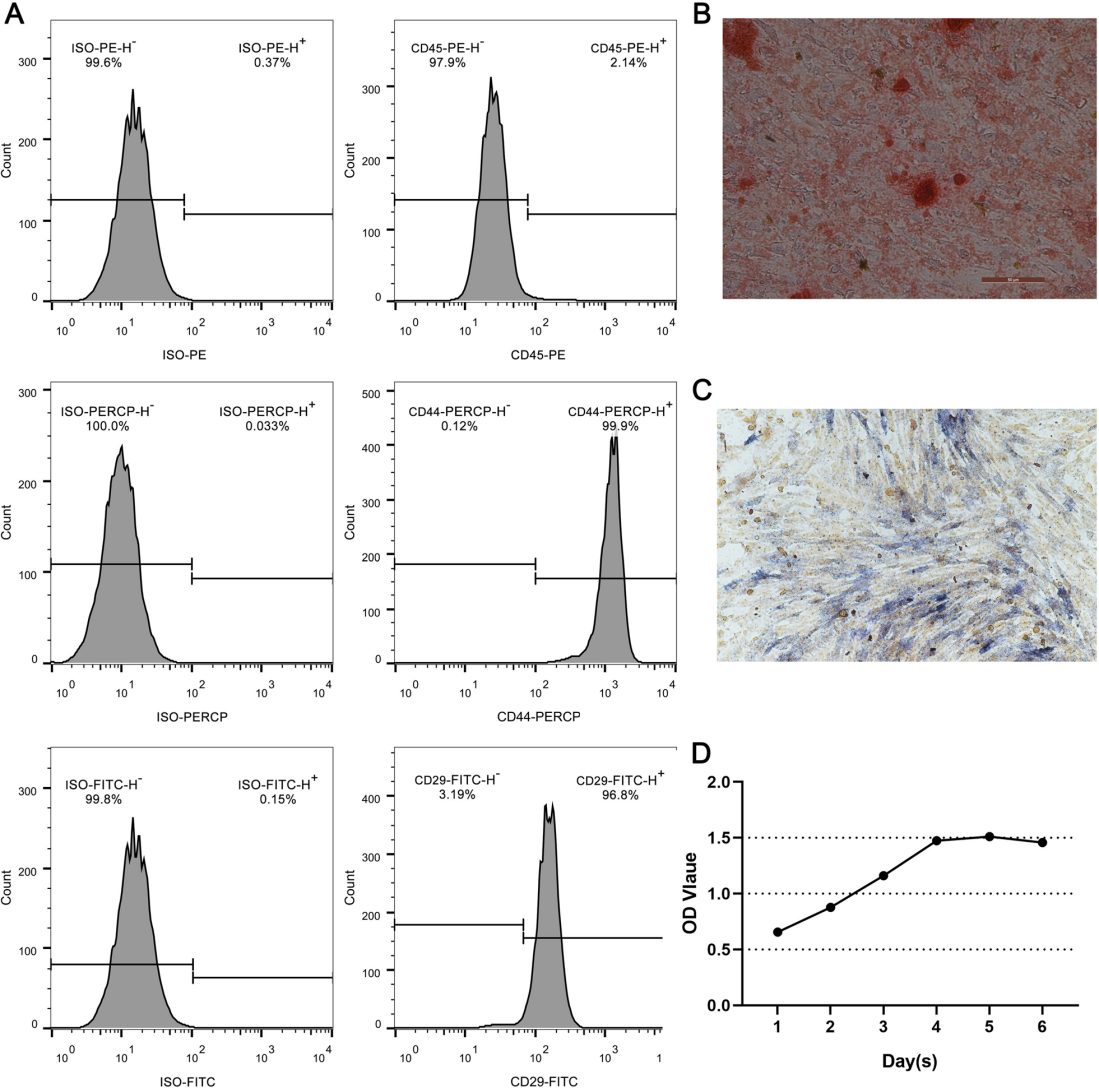
The identification of MSCs. **A**Flow cytometry detection results of MSCs. **B** Alizarin red staining was positive (inverted phase contrast microscope, × 10). **C** Alkaline phosphatase staining was positive (fluorescence microscopy, × 100). **D** The cell growth curve of MSCs

As shown in Fig. 1A-B, from the 9th day of culture adhesion, long spindle-shaped and triangular cells crawled out; the nuclei were round or ovoid, and the nucleoli were clear. After sub-culturing to the fourth generation, the cell fusion boundary became blurred; the cell body increased and its morphology gradually turned into a short spindle shape, polygon shape, and irregular shape; cells were arranged in parallel in a spiral or radial pattern and exhibited good growth. The flow cytometry results (Fig. 2A) showed that low expression of CD45 (2.140%) and high expression of CD29 (96.8%) and CD44 (99.9%). These results preliminarily indicated that the adherent cells isolated from the human alveolar bone in this experiment were BMSCs. In this experiment, following the osteogenic induction of MSCs for 14 days, alizarin red staining was performed; it was found that the mineralized nodules were scattered in orange and had different sizes. After osteogenic induction of MSCs for 7 days, ALP staining was performed; the active sites of ALP genes were bluish violet and the nuclei were red (see Figs. 2B and C).

#### CCK-8 proliferation test results

On days 1–2, the proliferation rate of the cultured MSCs was slow. On days 3–5, MSCs entered the exponential growth phase of massive fragmentation. The proliferation rate was observably accelerated, and the activity was enhanced compared with the previous two days. On day 5, the proliferation rate reached a peak and appeared to be stable, indicating that cell proliferation occurred rapidly in this phase. On day 6, cell growth plateaued (see Fig. 2D).

### Observation of MSCs’ attachment and adhesion on the surface of the titanium disk using SEM

**Fig. 3:**
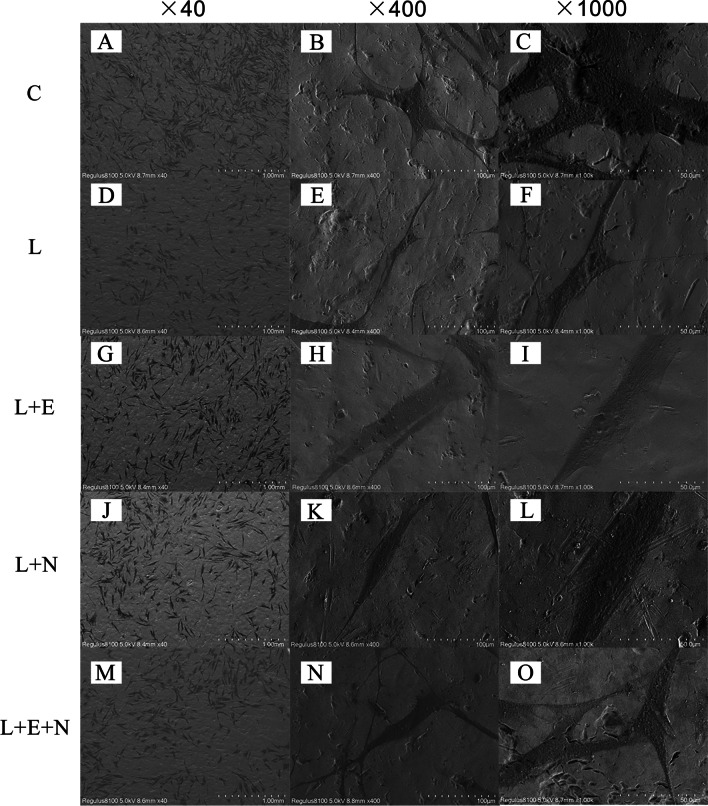
MSCs morphology on the surface of the titanium sample at 24 h after cell seeding

SEM was used to observe the morphology of the five groups of cells 24 h after inoculation onto a titanium disk (see Fig. 3). The cells in each group showed good growth and adhesion on the titanium disk and were in a tiled state. The cell density of group C was the highest. The cell in group C diameter was large with rich filopodia. The pseudopodia extended and were spread out evenly in all directions. Following amplification, the cell bodies were full, and the cells were connected by small synapses with good extension. Group L had the least amount of cells with only a few pseudopodia extending out and showing poor extension. The cell density of the L + E and L + N groups was slightly lower compared with group C. Only a few pseudopodia extended out in these two groups, and these were long and thin. The cell density of the L + E + N group was similar to that of the L + E and L + N groups; however, the cells in the L + E + N group were full and had more than 2 pseudopodia. The cells were in contact with one another through the pseudopodia, and the pseudopodia that extended out from the cell body were anchored onto the surface of the titanium disk.

### Alveolar bone marrow mesenchymal stem cell proliferation on the titanium disk

**Fig. 4:**
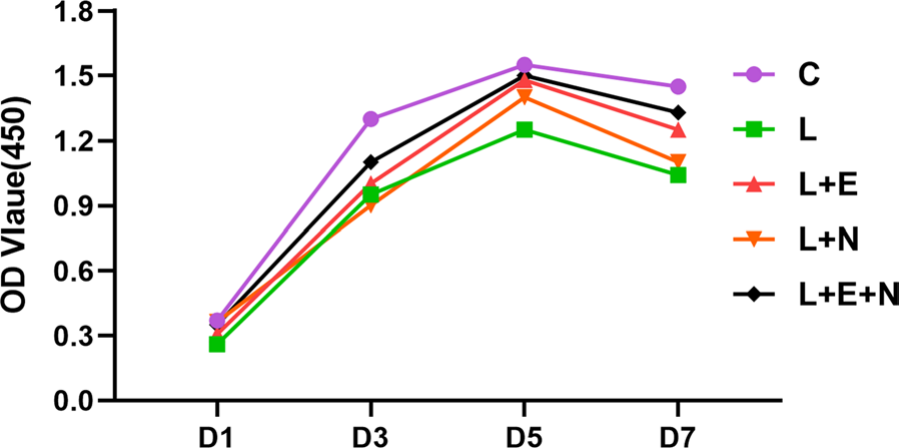
Cell proliferation in each group

The proliferation absorbance of each group of cells on the titanium disk was detected using the CCK-8 kit (see Fig. 4). The cell proliferation of each group showed a slow downward trend following an initial rise. On day 3, the OD value of the L, L + N, L + E, and L + E + N groups was smaller than that of group C (P < 0.05), but there was no significant difference between the L + N, L + E, and L + E + N groups and group L (P > 0.05). On day 5, the OD value of the L + N and L + E + N groups was greater than that of group L (P > 0.05), and there was no significant difference between the L + N, L + E, and L + E + N groups and group C. On day 7, the OD value of the L + E + N group was greater than that of group L (P < 0.05), and there was no significant difference between the L + N, L + E, and L groups (P > 0.05).

### Determining the mRNA expression level of the inflammatory cytokines

**Fig. 5:**
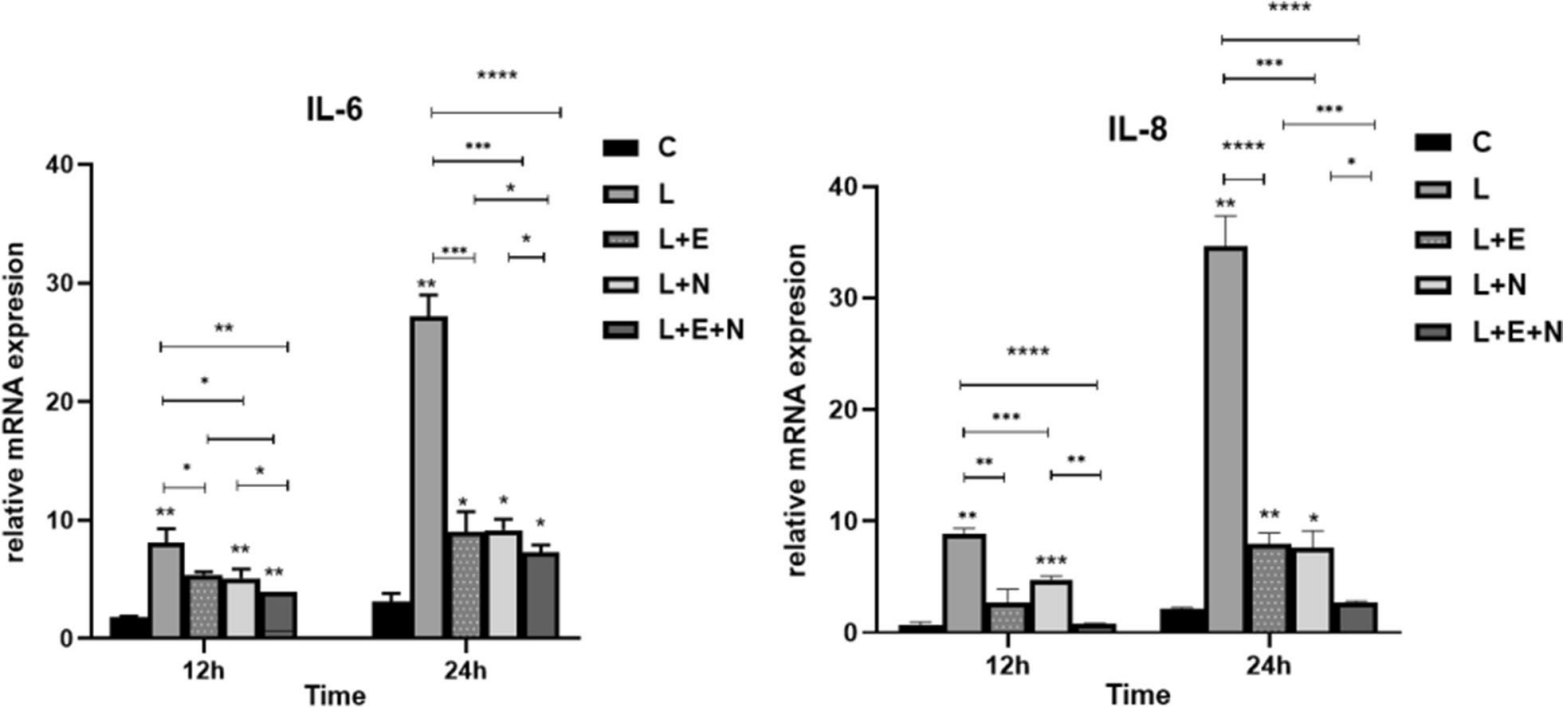
Expression of IL-6, IL-8mRNA in each group

The mRNA expression of BMSC inflammatory cytokines (IL-6 and IL-8) on the surface of the titanium disk in each group was detected at 12 and 24 h. As shown in Fig. 5, the expression level of inflammatory cytokines in each group increased with time. The 12 and 24 h test results showed that, compared with group C, the IL-6 mRNA expression level of groups L, L + N, L + E, and L + E + N was significantly up-regulated (P < 0.05). At 24 h, the IL-6 mRNA expression levels of the L + N, L + E, and L + E + N groups were lower than in group L, but the IL-6 mRNA expression level of the L + E + N group was significantly lower compared with group L (P < 0.0001). The 12 and 24 h test results of IL-8 mRNA showed that its expression level in groups L, L + N, and L + E was higher than in group C (P < 0.05), but there was no significant difference in IL-8 mRNA expression level between the L + E + N and C groups (P > 0.05). The IL-8 mRNA expression level of the L + E + N group was significantly lower than in group L (P < 0.0001).

### Determining the mRNA expression level of osteogenesis-related genes

**Fig. 6:**
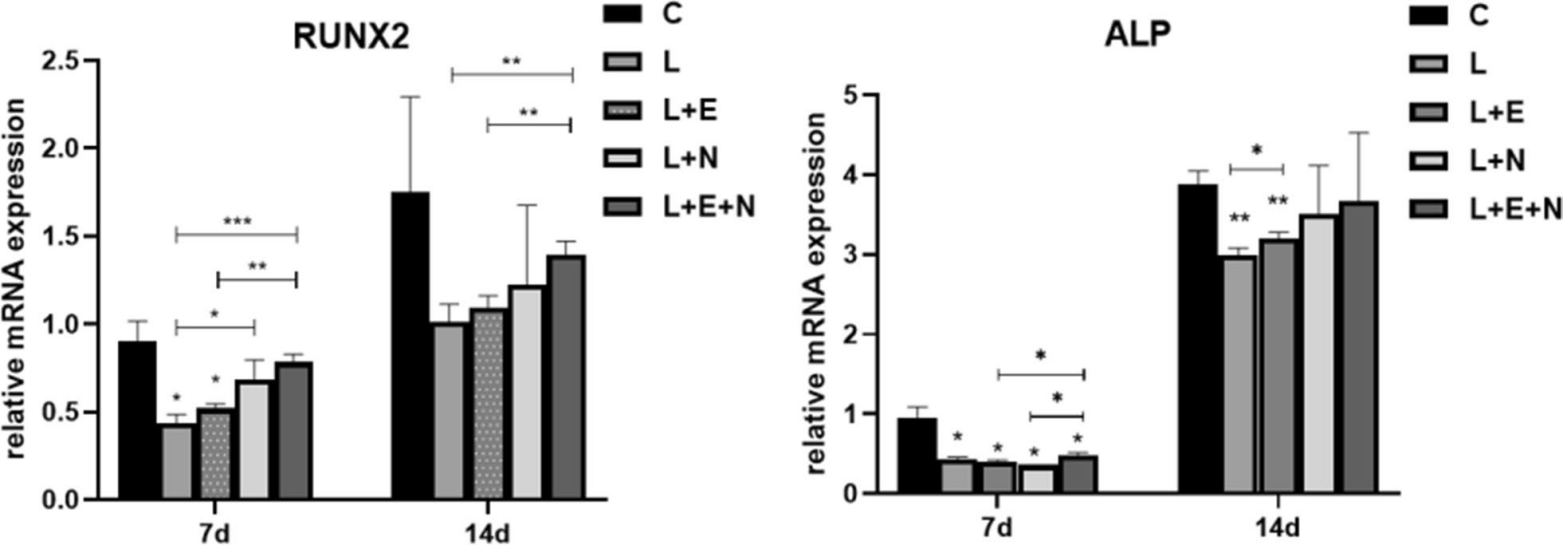
Expression of Runx2, ALP mRNA in each group

On days 7 and 14, the mRNA expression level of osteogenesis-related genes (ALP and RUNX2) in MSCs on the titanium disk was detected for each group. As shown in Fig. 6, the mRNA expression level of ALP and RUNX2 in the cells of each group increased with time. On day 7, the ALP mRNA expression level of the L, L + N, L + E, and L + E + N groups was lower compared with group C (P < 0.05). On day 14, there was no significant difference in the ALP mRNA expression level between the L + N and L + E + N groups and group C (P > 0.05), but the ALP mRNA expression level of the L and L + E groups was significantly lower than in group C (P < 0.01). On day 7, the RUNX2 mRNA expression level of the L + E + N group was significantly higher compared with group L (P < 0.001). On day 14, compared with group C, there was no significant difference in the RUNX2 mRNA expression level among the L, L + N, L + E, and L + E + N groups (P > 0.05), and the RUNX2 mRNA expression level of the L + E + N group was significantly higher compared with group L (P < 0.01).

## Discussion

Peri-implantitis is an infectious disease that occurs in the tissue around dental implants. It is characterized by the inflammation of tissue in the vicinity of an implant and the progressive loss of supporting bone [10].Both Er:YAG and Nd:YAG lasers apply to the treatment of peri-implantitis because of their anti-inflammatory and sterilizing effects, and their use can also promote bone healing [11].The Er:YAG laser can make the water molecules in the irradiation area absorb the laser energy and instantly evaporate, after which soft and hard tissue are melted by microblasting. The laser’s low penetrability and water misting effect reduce thermal injury to the tissue, which is beneficial in terms of supporting better bone healing.

The immediate high energy produced by an Nd:YAG laser causes bacterial solution vaporization, leading to cell-wall disintegration and, accordingly, sterilization and disinfection [12]. In addition, it has a biostimulation effect on cell activity and supports the proliferation of osteoblasts [13].

Some researchers have combined the two above-noted laser methods in the treatment of peri-implantitis. Compared with a traditional treatment method, Twinlight laser therapy can effectively reduce the sulcus bleeding index and the probing depth, thereby achieving better clinical efficacy [14].Meanwhile, Twinlight laser treatment contributes to improving cutting efficacy, shortens the operation duration, and promotes the improvement of patients' intraoperative comfort level and postoperative satisfaction, giving it high clinical application and promotional value. However, there are currently few reports on the mechanism of action involved in Twinlight laser treatment.

### The acquisition of MSCs and establishing the peri-implantitis model

MSCs derive from oral alveolar bone and have multiple differentiation potentials. They can be differentiated into, e.g., osteoblasts, adipocytes, and endothelial cells, and contribute to repairing hard and soft tissue around the implant [15].Bone marrow mesenchymal stem cells are an important source of osteoblasts and are often used as core cells for repairing local bone, gristle, and myelogenous adipose tissue. These cells can be adhered to a titanium surface and activated in the osteogenesis and osseointegration phases [16]. There are two ways in which to obtain inflammatory MSCs, i.e., the in-vitro induction of an inflammatory microenvironment and culturing in the periodontia of patients with peri-implantitis whose implants must be removed. The major pathogenic bacterium of peri-implantitis is a gram-negative anaerobic bacterium. Liposaccharides, as one of the strongest virulence factors, can activate a series of host cells to release proinflammatory cytokines and inflammatory mediators, thereby directly or indirectly causing inflammation. Through PCR tests, Lu et al. [17] found that LPSs could up-regulate the expression of inflammatory cytokines (IL-6 and IL-1β) and transforming growth factor beta in BMSCs. Yuli Wang et al. [18] used 150-μg/L LPS osteogenesis to establish a BMSC inflammation model. By conducting a western blot analysis, the authors found that LPSs could lower the content or relative expression level of both bone morphogenetic protein-2 and the ALP gene.

The above studies indicate the feasibility of establishing an in-vitro inflammation model. Compared with other modeling methods, this approach lacks a bacterial effect; however, LPSs can rapidly induce the host’s immune response in a manner similar to the endotoxins of some pathogenic bacteria present in peri-implantitis [19].In the current study, a concentration of 1-μg/ml LPSs was used. By referring to domestic and foreign literature and combining existing preliminary experiments with the results of the current study, the authors found that this concentration was perfectly suitable and could cause inflammatory injury in MSCs without causing significant cell death. Hence, it was concluded as a reasonable induction dose. In this study, the significant elevation in the expression level of IL-6 and IL-8 mRNA in group L verified this.

Because of its excellent mechanical properties and biocompatibility, titanium has become a mainstay material for use in dental implants. Many studies have shown that treating the surface of titanium with an Er:YAG laser can effectively enhance osseointegration between titanium disks and cells, without damaging the surface [20].Hauser et al. [21] compared the surface of a titanium implant treated with an Er:YAG laser (63.69 J/cm^2^) with the same surface in a control group without any treatment and found that laser treatment was beneficial to the colonization and proliferation of osteoblasts. Hani et al. [22] compared the removal effect of an Er:YAG laser, titanium brush, and a carbon fiber tip on the biofilm of implants with a titanium surface. The researchers created a titanium plate for 8 individuals who wore the device for 72 h to form biofilms. The titanium plate was then removed. The 8 individuals were then randomly divided into 4 treatment groups. The residual biofilms were observed under a fluorescence microscope, and image analysis software was used for the quantitative analysis of the residual biofilms. It was concluded that for titanium surfaces without threads, Er:YAG laser therapy was an effective approach for reducing bacterial biofilms on the titanium plate. Guo Zehong et al. [23] inoculated osteoblasts onto laser-etched and smooth titanium disks and observed cell adhesion microscopically. After inoculation, immunofluorescence staining was performed, and a laser scanning confocal microscope was used to observe the cytoskeleton and morphology. It was concluded that, following laser etching treatment of the titanium surface, the surface morphology was controllable and the microstructure of the titanium surface could be increased without causing cytotoxicity, thereby promoting the early proliferation of human osteosarcoma (MG63) cells.

In this study, an Er:YAG laser (MSP, 60 mJ, 20 Hz, 1.2 W; water, 4; gas, 4) was used to treat a titanium disk surface to simulate debridement in the clinical treatment of peri-implantitis. Cells treated using different methods were inoculated onto the titanium disk surface, and SEM showed that inflammatory MSCs could grow and extend on the titanium disk’s surface. Additionally, qRT-PCR results showed that compared with group C, the mRNA expression level of inflammatory cytokines (IL-6 and IL-8) rose significantly in group L (P < 0.01), indicating the successful establishment of a peri-implantitis model.

### The effect of Twinlight laser treatment on titanium surface proliferation and the morphology of inflammatory MSCs

Scanning electron microscopy indicated that cells treated with a variety of methods grew and adhered well to the titanium disks. Compared with the LPS induction group, the cells in group C were the densest. Compared with the L + E and L + N groups, the MSCs in the L + E + N group had richer filopodia; additionally, the slender filopodia of MSCs could potentially extend continuously to seek more suitable adhesion sites. The proliferation capacity of MSCs on the titanium disk’s surface was tested by CCK-8, and the results showed that the absorbance proliferation trend of MSCs in the five groups on the titanium disk’s surface was consistent with the growth trend of the cells in the 6-well plate, presenting a trend showing a slow decline after an initial rise. Days 1–3 represented a rapid growth period and days 5–7 a gentle growth period; the reason for this may have been because cell growth space had been reduced. Hou et al. [24] stimulated the BMSCs of rats with a 5.0 J/cm^2^ diode laser; MTT test results showed that, compared with the C group, the diode laser significantly promoted BMSCs’ proliferation, growth factor secretion, and differentiation. On days 5–7, the absorbance value of the L + N, L + E, and L + E + N groups in the present study that had been laser-treated was higher compared with group L, verifying that laser treatment could promote inflammatory cell proliferation.

Concerning absorbance value, on days 3–7, in the L + E + N group, this value was higher compared with the L + N and L + E groups, while the value in the L + E group was higher compared with that of the L + N group. It was thus concluded that Er:YAG laser treatment had a better effect compared with Nd:YAG laser treatment in terms of promoting the proliferation capacity of inflammatory MSCs. Twinlight laser treatment had a better effect compared with using an Er:YAG or Nd:YAG laser on their own in terms of promoting the proliferation capacity of inflammatory MSCs.

### The effect of Twinlight laser treatment on the inflammatory response of inflammatory MSCs

Peri-implantitis is typically caused by the overexpression of proinflammatory cytokines and inflammatory mediators in a local microenvironment. According to the pathogenesis of peri-implantitis, reducing the production of proinflammatory cytokines, inhibiting alveolar resorption, and promoting new bone formation may be effective ways of treating this condition. Interleukin-6 is a cytokine produced by activated T-cells and fibroblasts, which are involved in the development of inflammation and influence the growth and differentiation of various cells through their pleiotropy. Interleukin-8 is an inflammatory chemokine that promotes an inflammatory response, thus stimulating angiogenesis and facilitating mitosis, which is closely linked to the occurrence and development of various diseases [25]. Chu Tienan et al. [26] studied patients receiving implant repair therapy and found that inflammatory cytokines, such as IL-6, IL-8, and tumor necrosis factor alpha (TNF-α), could be detected in gingival crevicular fluid with inflammation manifestation in gingival implant tissue. Jin Xiaolan et al. [27] detected TNF-α, IL-8, IL-1β, and IL-6 in the supernatant of inflammatory and normal cells using an ELISA method, and concluded that low-level laser irradiation could protect against the inflammatory damage of LPS-induced human periodontal ligament fibroblasts. Huang et al. [28] found that the diode laser reduced the expression of inflammatory markers IL-6 and IL-1 in MG63 induced by LPSs and improved cell proliferation capacity.

The current experiment found that, as time passed, the expression level of inflammatory cytokines in the cells of each group increased, and compared with group C, the expression level of IL-6 and IL-8 mRNA in group L rose significantly (P < 0.01). Compared with group L, the expression levels of IL-6 and IL-8 mRNA in the L + N, L + E, and L + E + N groups were lower; however, the decline in the L + E + N group was the most significant (P < 0.0001), indicating that Er:YAG, Nd:YAG, and Twinlight laser treatments could inhibit the expression of inflammation-related genes in inflammatory MSCs induced by LPSs on a titanium disk and reduce inflammatory injury; however, Twinlight laser treatment of inflammatory MSCs could more effectively inhibit the expression of inflammatory cytokines.

### The effect of Twinlight laser treatment on the osteogenic differentiation ability of inflammatory MSCs

Among the multiple differentiation abilities of BMSCs, osteogenic differentiation is an important function for repairing tissue defects around implants. The genes related to osteogenic differentiation include the ALP and RUNX2 genes. The former is indispensable in the osteoblast mineralization process. When there is no increase in the level of inorganic phosphates in the components of bone mineral phases, the level of ALP will be high. Thus, ALP activity is considered an early indicator of osteoblast differentiation [29]. Sun et al. [30] used LPSs in 1-μg/ml *Escherichia coli* to stimulate the BMSCs of rats to establish an inflammation environment; the researchers found that LPSs could significantly inhibit ALP expression and osteogenic differentiation in cells, thus indicating that inflammation reduced the ALP expression level of BMSCs in rats. Liu et al. [31] found that after adding low-concentration LPSs (100 μg/L) to an osteogenic medium, ALP gene activity in BMSCs declined. The researchers concluded that in the inflammatory response induced by LPSs, the increased expression of proinflammatory cytokines generated an inhibiting effect on the differentiation of osteoblasts.

The RUNX2 gene is an important transcription factor for osteoblast differentiation and bone formation, and also functions as a mark of osteoblast differentiation. It can activate the expression of type-I collagen, OC, and other downstream factors in the osteogenic differentiation regulatory network, promote osteogenic differentiation, accelerate extracellular matrix deposition, and form bone tissue [32]. Luo et al. [33] used LPS to stimulate the BMSCs of rats; they found that LPSs inhibited the expression level of factors (ALP and RUNX2) related to osteogenic differentiation and type-I collagen. Wang et al. [34] assessed the effect of low-level laser therapy (LLLT) with different energy intensities on the proliferation and osteogenic differentiation of BMSCs under healthy and inflammatory microenvironments. They concluded that LLLT with a density of 8 J/cm^2^ could promote BMSC proliferation and osteogenesis. Bai et al. [35]. co-cultured in vitro BMSCs and human umbilical vein endothelial cells and found that LLLT enhanced vascular bone regeneration through the coupling of angiogenesis and osteogenesis. Reactive oxygen species regulate hypoxia-inducible factor 1 alpha may be key to the formation of H-shaped blood vessels and the enhancement of osteogenic differentiation in BMSCs. This enhanced angiogenesis further promoted bone repair and regeneration and provided new perspectives on the role of LLLT in fracture healing and tissue engineering strategies. Furthermore, the effect of LLLT on cell proliferation, angiogenesis, and osteogenesis was assessed. The results of this experiment showed that, as the time of osteogenesis-induced differentiation increased, the expression of osteogenesis-related genes, i.e., ALP and RUNX2 mRNA, increased. On days 7 and 14 of osteogenesis-induced differentiation, the expression level of ALP and RUNX2 mRNA in the L, L + N, L + E, and L + E + N groups was lower than in group C, which was consistent with the findings of Bandow, indicating that LPSs could effectively inhibit the osteogenic differentiation ability of MSCs. On day 14, the expression levels of ALP and RUNX2 mRNA in the L + N, L + E, and L + E + N groups were higher than in group L, but the expression level of RUNX2 mRNA in the L + E + N group was significantly higher than in group L (P < 0.01). There was no significant difference in the expression level of ALP and RUNX2 mRNA between the L + N, L + E + N, and C groups (P > 0.05), indicating that Er:YAG, Nd:YAG, and Twinlight laser treatment could promote the expression of inflammation-related genes in inflammatory MSCs induced by LPSs on a titanium disk, and fulfill a specific osteogenic role. It is noted, however, that Twinlight laser treatment of inflammatory MSCs could more effectively promote the expression of osteogenesis-related Al-BMSC genes.

The results of the current study showed that Twinlight laser treatment could promote cell proliferation, down-regulate the mRNA expression of inflammatory cytokines, and effectively enhance the osteogenic differentiation of cells on a titanium disk surface. This study provides a theoretical basis for Twinlight laser adjuvant therapy in cases of peri-implantitis. Since this experiment was performed using an in vitro model, it is necessary to further improve animal-based experiments and clinical studies in follow-up experiments for verification purposes.

## Conclusion

Treatments using Er:YAG and Nd:YAG lasers had an inhibiting effect on the expression of inflammation-related genes in inflammatory MSCs on a titanium disk surface, and Twinlight laser treatment could more effectively inhibit the expression of inflammatory BMSC cytokines. Additionally, Er:YAG and Nd:YAG laser treatments promoted the expression of osteogenesis-related genes in inflammatory MSCs on a titanium surface, and Twinlight laser treatment could more effectively promote the expression of osteogenesis-related BMSC genes.

## Data Availability

The datasets used and/or analysed during the current study available from the corresponding author on reasonable request.
